# Does perioperative hydrocortisone reduce morbidity after pancreatoduodenectomy? A propensity score matched analysis

**DOI:** 10.1007/s13304-025-02280-4

**Published:** 2025-07-20

**Authors:** O. Radulova-Mauersberger, F. Oehme, N. Mibelli, C. Teske, G. Krause-Jüttler, J. Weitz, M. Distler, S. Hempel

**Affiliations:** 1 Department of Visceral-, Thoracic and Vascular Surgery, Faculty of Medicine and University Hospital Carl Gustav Carus, Technische Universität Dresden, Dresden, Germany; 2 National Center for Tumor Diseases (NCT/UCC), Dresden, Germany; 3 German Cancer Research Center (DKFZ), Heidelberg, Germany; 4 Faculty of Medicine and University Hospital Carl Gustav Carus, Technische Universität Dresden, Dresden, Germany; 5 Helmholtz-Zentrum Dresden - Rossendorf (HZDR), Dresden, Germany

**Keywords:** Pancreatoduodenectomy (PD), Postoperative pancreatic fistula (POPF), Postpancreatectomy pancreatitis (PPAP), Postoperative hyperamylasemia (POH), Hydrocortisone (HC)

## Abstract

The use of steroids in pancreatic surgery may reduce complications through the anti-inflammatory potential. We aimed to evaluate the impact of hydrocortisone (HC) on postoperative morbidity after pancreatoduodenectomy (PD). The data were retrospectively extracted from a prospective clinical database. Complicatons after PD with 100 mg HC intravenously (i.v.) for 48 h and a corresponding propensity score-matched cohort without HC were evaluated by using logistic regression analyses. We analyzed 691 patients who underwent PD over a period of ten years. After propensity score matching, 220 patients (110 patients in each group) remained. Postoperative complications were comparable in both groups (*p* = 0.56). Major complications (CDC ≥ 3) did not differ significantly, *n* = 44 (40%) for non-HC *n* = 45 (40.9%) and HC group (*p* = 0.89). There was no difference (*p* = 0.88) in the POPF incidence, non-HC, *n* = 31 (28.2%) and HC group, *n* = 30 (27.3%). After stratifying for fistula risk score (FRS), results remained equal and no difference was observed in CDC ≥ 3 in the low (*p* = 1), intermediate (*p* = 0.82) and high-risk (*p* = 0.74) group. HC has been suggested to be effective in reducing postoperative morbidity, but the evidence is controversial. The benefit of HC in reducing postoperative complications after pancreatoduodenectomy cannot be supported in the present study.

## Introduction

Pancreatic resection is the only curative treatment for carcinoma, and outcomes and perioperative management have improved considerably in recent years [22]. Both clinical and epidemiological research have contributed to further improve intra- and perioperative protocols and to gain and improve knowledge about the predictors and therapy of complications. Postoperative complications after pancreatic surgery rating 30–50% are still remarkable [10, 22]. Among the most common are postoperative pancreatic fistula (POPF), postpancreatectomy hemorrhage (PPH) and delayed gastric emptying (DGE). Recently, postpancreatectomy pancreatitis (PPAP) has been increasingly reported and studied as a separate complication. Following the approach adopted for POPF, PPAP has been graded into three severe grades based on its clinical course. It is characterized by inflammation of the pancreas remnant and may cause symptoms such as abdominal pain, nausea, vomiting and fever [27]. However, there is still a lack of evidence related to the pathogenesis and clinical presentation of PPAP. The etiology is multifactorial and is considered to follow surgical trauma, ischaemia–reperfusion injury, and the release of inflammatory cytokines [13]. Furthermore, POPF is also frequently reported to occur in the case of PPAP, and although no clear clinical and diagnostic borderline has yet been defined, it is widely suggested that these are two different entities, and pancreatitis is triggered by an inflammatory reaction in the pancreas. The majority of data reported are related to postoperative hyperamylasemia (POH) as the early manifestation of PPAP and shows that this condition increasingly affects the postoperative course, which requires effective treatments to reduce its incidence and severity*.*

Steroids are known for their potential beneficial effect in reducing postoperative inflammation and recent studies suggest this affects PPAP as well. Dexamethasone, which is a long-acting steroid (up to 72 h) with an anti-inflammatory effect, and HC as a short-acting (8–12 h) synthetic steroid that binds to glucocorticoid receptors, suppressing inflammatory cytokines were found to reduce postoperative inflammatory response and postoperative complications [8, 13, 27].

The evidence of the effectiveness is still very limited and current findings are somewhat controversial thus the use of steroids in pancreatic surgery requires further evaluation. Therefore, we evaluated the outcome after PD, following the onset of perioperative HC administration in our institution, aiming to assess the potential impact of HC on postoperative morbidity, POPF and POH/PPAP.

## Methods

### Patient cohort

The study protocol was approved by the local ethics committee of the Technische Universität (TU) Dresden (decision number BO-EK-158042024). Data is reported in accordance with the Strengthening the Reporting of Observational Studies in Epidemiology (STROBE) guidelines [29]. A single-center propensity score-matched analysis was performed for this study. The population studied included all pancreatic head resections carried out between 2012 and 2022 at the Department of Visceral, Thoracic and Vascular Surgery, University Hospital Carl Gustav Carus, Technical University of Dresden, Germany. Since 2019, all patients with pancreatic resections have received perioperative HC according to the clinical standard operating procedures (SOPs). An existing database, which included all PD`s in the reported period was used to define the corresponding propensity score-matched patient cohort.

Inclusion criteria for the study were pancreatic head resection (either pylorus-preserving pancreatic head resection [PPPD], or classic pancreaticoduodenectomy [cPD]) for pancreatic and periampullary carcinoma, neuroendocrine tumors, cystic lesions of the pancreas, chronic pancreatitis or benign tumors.

**Fig. 1:**
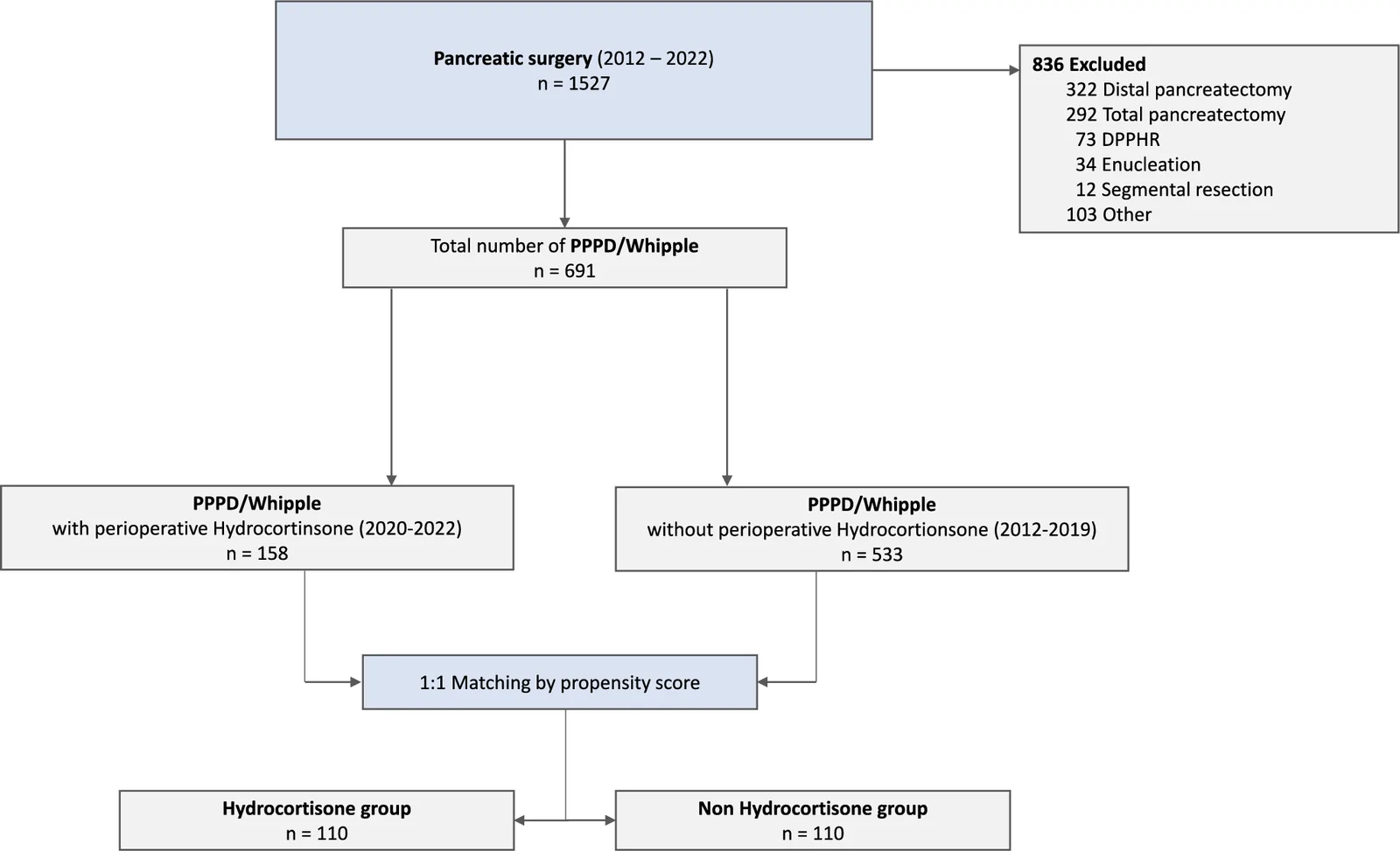
Flowchart of the retrospective data analysis

Baseline patient characteristics included age, gender, BMI, preoperative biliary stenting, history for diabetes, nicotine and alcohol consumption and physical status according to the ASA score. The perioperative administration of HC was assessed and used for cohort selection. Using the baseline patient characteristics, the patient cohorts were randomly matched (see below “*statistical analysis*”) during the analyzed period and all medical records were collected from electronic patient files (Fig. 1).

### Hydrocortisone perioperatively

Since January 2019, all patients scheduled for PD were administered HC 100 mg intravenously (i.v.) during anesthesia induction. In case of intermediate or high pancreatic fistula risk, according to FRS ≥ 3, HC was continued postoperatively every 8 h with 100 mg i.v. for 2 days (48 h). This perioperative pharmacotherapy protocol was adopted in the clinical SOPs after the promising results of the Finnish randomized controlled trial by Laanien et al [13].

### Surgical procedure and postoperative management

Both minimally invasive robotic and open surgical procedures were included in the study. Only cases where the reconstruction was performed as an end-to-side duct-to-mucosa pancreaticojejunostomy were included. The pancreas transection was carried out using a stapler or scalpel for robotic and open PD, respectively. The reconstruction was performed further with an end-to-side hepaticojejunostomy, performed with either interrupted or continuous suture and placed approximately 15 cm distal to the pancreatic anastomosis followed by an end-to-side duodenojejunostomy or gastroenterostomy with two layers of continuous sutures.

Drainage management followed the clinic standards, and drainage was placed if FRS ≥ 3 (intermediate or high) [6]. When drainage was applied, amylase and lipase activity were routinely measured in drainage fluid at POD 1 and 3 to determine POPF evidence. According to reported evidence, if the level of amylase was less than 85 µmol/L (less than 5000 U/mL) and the volume in 24 h less than 800 mL of serous fluid, the drainage was removed on postoperative day 3 [19].

After surgery, a CT scan of the abdomen was considered for patients with CRP > 140 mg/L on postoperative day (POD) 4 or based on clinical findings. If fluid collection was found and a drainage was feasible, an interventional procedure (eg, CT-guided drainage or endoscopic transgastric drainage) was performed. In these cases, a POPF B or C depending on the clinical course and amylase level in drainage fluid, was recorded.

We used the Clavien-Dindo classification (CDC) to record postoperative morbidity, which was defined as any complication occurring after the index surgery during the length of hospital stay [9]. The ISGPS definitions and grading were used for POPF, post-operative hemorrhage (PPH) and delayed gastric emptying (DGE) [5, 30, 31]. Wound infections were documented if they were clinically relevant and led to a prolongation of the hospital stay or recurrent surgical revisions. The definition of sepsis was in accordance with the international guidelines [14]. We documented the postoperative hyperamylasemia (POH) to be the initial level of PPAP in accordance with the new definition [3, 18]. Pancreatic texture was described as hard or soft according to the surgeon’s intraoperative assessment. The fistula risk score was calculated according to histology, pancreatic texture, pancreatic duct diameter, and the intraoperative blood loss as defined by Callery et al., and graded into negible/low (0–2), intermediate (3–6), and high (7–10) risk levels [6]. Further the ISGPS classification on risk of POPF grading A-D according to the pancreatic texture and pancreatic duct diameter was assessed for both cohorts to strengthen their comparability [26].

### Study endpoints

The primary endpoint of the study was to evaluate whether HC reduces postoperative morbidity with a focus on POH/PPAP. The secondary endpoint was to assess the effect of HC on subgroups of patients after FRS stratification. Propensity score matching analysis, as a statistical method to reduce bias, was applied to compare patients with similar characteristics [21].

### Statistical analysis

Data were analysed using SPSS version 26 software (IBM Corp., Armonk, NY, USA). The normality of continuous data was assessed by using Kolmogorov–Smirnov test and through examining the frequency distributions. Variance homogeneity was analyzed using Levene’s test.

The two patient cohorts were matched as a first step. We conducted a propensity score matching between the HC and non-HC groups using the nearest neighbour method in a 1:1 ratio. A threshold of < 0.2 was set for the width of the propensity score deviation. The Cohens* d*-value was calculated before and after matching to show the effectiveness of the matching process (Table 2).

The variables used in the matching process were age, gender, ASA, BMI, histology, alcohol consumption, smoking, diabetes, pancreas texture, pancreatic duct diameter, FRS and ISGPS ABCD risk classification for POPF. Standardized mean deviation analysis was used to detect residual discrepancies after matching.

In order to harmonize the cohorts, a stepwise approach for the matching procedure and the use of additional variables to avoid overfitting was considered. Principal component analysis was used to convert the correlated variables into independent principal components [15]. After matching within a given FRS profile and ABCD ISGPS-POPF risk score, a wide range of patients with varying FRS scores (e.g., intermediate-risk patients in the range of 3–6) remained. Parameter such as blood loss, pathological differentiation, pancreatic duct diameter and texture differed and contributed to heterogeneity within the groups. To avoid suboptimal between-group comparisons, separately a stepwise matching by pancreatic texture and pancreatic duct diameter was performed to ensure that patients were matched with respect to their individual fistula risk. Finally, smaller patient cohorts, which, however, provided better comparability due to their more homogeneous characteristics were included in the analysis [15].

For categorical variables, the chi-squared test or Fisher’s exact test was used as a competitive analysis to compare baseline characteristics between the HC and non-HC groups. For continuous variables, Student’s* t*-test or Mann–Whitney* U* test was used where appropriate. The results were reported as median and interquartile range (IQR).

For the entire study, a *p* value of < 0.05 was considered the threshold for statistical significance. During the analysis, missing data were treated as missing at random for the appropriate analysis. In this way, a complete analysis of the cases was performed, and some patients were excluded.

## Results

### Description of the study cohort and the baseline characteristics

In total, 1527 patients who underwent pancreatic surgery between 2012 and 2022 were included in the study. Thereof, 691 patients underwent a PD (PPPD or cPD) and 158 patients in this group were administered HC perioperatively. Based on data from two RCT`s from Finland, this procedure became our institution`s standard in 2019 [13, 27]. After matching the HC with the non-HC group (*n* = 533), the cohorts were comparable and differed in none of the baseline characteristics significantly (Table 1).

**Table 1:** Characteristics of unmatched cohorts

	Non-HC	HC	*p* value	COHEN`S d
Patients [*N* (%)]	530	158		
Females [*N*(%)]	221 (41.7)	61 (38.6)	0.48	0.07
Males [*N* (%)	309 (58.3)	97 (61.4)	0.57	0.09
BMI [KG/M^2^] (IQR)	24.9 (22.6–28)	25.2 (22.3–28.4)	0.8	0
Pancreatic main duct diameter [mm] (IQR)	4 (3–5)	3 (3–4)	**0.03**	0.55
FRS [N] (IQR)	4 (2–6)	4 (3–6)	**0.03**	0.54
Soft pancreas [*N* (%)]	253 (48.7)	101 (65.2)	** < 0.01**	0.37
High risk FRS [*N* (%)]	77 (14.9)	21 (13.5)	0.66	0.31
ISGPS - POPF risk score [*N* (%)]				
GRADE A	175 (33.1)	37 (23.7)	** < 0.001**	0.13
GRADE B	57 (10.8)	25 (15.8)		
GRADE C	177 (33.3)	50 (31.6)		
GRADE D	121 (22.9)	46 (28.9)		
FRS [N (%)]				
0	25 (4.8)	1 (0.6)	** < 0.01**	0.18
1	68 (12.9)	8 (5.1)		
2	74 (13.9)	15 (9.6)		
3	80 (15.1)	38 (24.0)		
4	68 (12.9)	27 (17.1)		
5	66(12.4)	26 (16.7)		
6	69(13.1)	22 (14.1)		
7	50 (9.5)	15 (9.6)		
8	21 (3.9)	4 (2.6)		
ASA score [*N* (%)]				
1	18 (3.4)	0	0.37	**0.01**
2	207 (39.1)	75 (47.4)		
3	300 (56.5)	79 (50)		
4	5 (0.9)	4 (2.6)		
Blood loss [ML] (IQR)	500 (350–800)	500 (350–750)	0.28	0.09
Duration [MIN] (IQR)	349 (292–419)	381 (330–446)	** < 0.01**	0.2

*Non-HC* non-hydrocortisone, *HC* hydrocortisone, *IQR* interquartil range, *ISGPS* international study group for pancreatic surgery, *FRS* Fistula risk score, *POPF* postoperative pancreatic fistula, **numbers in bold show significance if p<0,05**

Both cohorts consisted of 65 (59.1%) male and 45 (40.9%) female patients with a median age of 69.5 (IQR, 62–77) and a median BMI score of 25 kg/m^2^ (IQR, 22–28). Most patients were operated on for adenocarcinoma (49.3%). The histological findings did not differ significantly between the two cohorts and were comparable (*p* = 0.2). The other matching parameters ASA (*p* = 0.69), smoking (*p* = 0.31) and alcohol consumption (*p* = 0.88) were also not significantly different after matching.

### Matched cohorts’ comparability according to POPF-related perioperative parameters

While focusing on risk factors for POPF, we analyzed if there is a significant difference between both cohorts to ensure the comparability regarding the endpoints of the study. The duration of surgery was similar for both cohorts (*p* = 0.49). Vascular resection (*p* = 0.45) and blood loss (*p* = 0.36) also did not differ significantly. The type of reconstruction was the only parameter the two groups differed, as there were more PPPD patients in the cohort without HC (78.2% vs. 59.1%). On the contrary, in the HC group there were more cPD patients (40.9% vs. 24%), which resulted in a significant difference (*p* < 0.01). Both groups were comparable within the matching variables POPF, pancreatic texture, FRS, ISGPS-ABSCD classification and pancreatic duct diameter (Table 2).

**Table 2:** Characteristics of matched cohorts

	Non-HC	HC	*p* value	COHEN`S d
Patients [*n* (%)]	110	110		
Females [*n* (%)]	45 (40.9)	45 (40.9)	1	0
Males [*n* (%)	65 (59.1)	65 (59.1)	1	0
BMI [kg/m^2^] (IQR)	25 (22.4–27.8)	24.7 (22.3–27.7)	0.69	0
Median age [YEARS] (IQR)	67.5 (60.3–76)	68 (61.1–75)	0.78	0
Pancreatic main duct diameter [mm] (IQR)	3 (3–4)	3 (3–4)	0.82	**0.02**
FRS [*n*] (IQR)	4 (3–6)	4 (3–6)	0.78	**0.03**
SOFT PANCREAS [*n* (%)]	58 (52.7)	59 (62.7)	0.13	0.22
HIGH RISK FRS [*n* (%)]	15 (13.6)	13 (11.8)	0.69	0.1
ISGPS - POPF RISK SCORE [*n* (%)]				
GRADE A	28 (25.5)	24 (21,8)	0.07	0.23
GRADE B	19 (17.3)	17 (15.5)		
GRADE C	42 (38.2)	31 (28.2)		
GRADE D	21 (19.1)	38 (34.5)		
FRS [*n* (%)]				
0	4 (3.6)	0		**0.04**
1	9 (8.2)	6 (5.5)		
2	11 (10)	12 (10.9)		
3	16 (14.5)	25 (22.7)		
4	20 (18.2)	17 (15.5)		
5	18 (16.4)	21 (19.1)	0.5	
6	17 (15.5)	16 (14.5)		
7	12 (10.9)	11 (10)		
8	3 (2.7)	2 (1.8)		
ASA SCORE [*n* (%)]				
1	4 (3.6)	3 (2.7)	0.45	**0.04**
2	42 (38.2)	50 (45.5)		
3	62 (56.4)	56 (50.9)		
4	2 (1.8)	1 (0.9)		
Blood loss [ml] (IQR)	500 (300–800)	500 (400–713)	0.36	0.12
Duration [min] (IQR)	356 (305–415)	380 (330–444)	0.26	0.14

*IQR* interquartil range, *FRS* fistula risk score, *cPD* whipple procedure, *PPPD* pylorus preserving pancreatoduodenectomy, *ISGPS* international study group for pancreatic surgery, numbers in bold show significance if p<0.05

### Outcome analysis

Between the matched cohorts, there was no significant deviation in postoperative complications. Regarding POPF (28.2% vs. 27.3%; *p* = 0.88), POH/PPAP (48.6% vs. 54.1%, *p* = 0.42) and PPH (17.3% vs. 15.5%, *p* = 0.72), the results in both groups were similar. The clinically relevant complications summed up as CDC ≥ 3 also did not differ significantly (*p* = 0.89). Rescue pancreatectomy which was performed exclusively in patients with CR-POPF and PPAP grade C, showed no significant difference between the two groups.

In-hospital mortality (1.8% vs. 9.1%; *p* = 0.03) and the length of ICU stay (IQR, 2–4.3 vs. IQR, 1–3; *p* = 0.05) were found to be significantly different, favoring the HC group. Further, there was a trend for more frequent events of DGE in the non-HC group (36.4 vs. 24.5%, *p* = 0.06) and for prolonged hospital stay in the same group (IQR, 13.8–27.5 vs. IQR, 11.8–27.3; *p* = 0.08), but no significant difference was found. Results are outlined in Table 3.

**Table 3:** Results in matched cohort

	Overall	Non- HC	HC	*p* value
Patients [N (%)]	220	110	110	
Length of hospital stay (DAYS) (IQR)	19 (13–27)	21.5 (13.8–27.5)	18 (11.8–27.3)	0.08
Length of ICU stay (DAYS) (IQR)	2 (1–4)	3 (2–4.3)	1 (1–3)	0.05
Postoperative inhouse complication [*N* (%)]				
COMPLICATION [*n* (%)]	154 (70)	75 (68.2)	79 (71.8)	0.56
CDC ≥ 3 [*n* (%)]	89 (40.5)	44 (40)	45 (40.9)	0.89
CR POPF [*n* (%)]	61 (27.7)	31 (28.2)	30 (27.3)	0.88
POH/PPAP [*n* (%)]	110 (51.4)	51 (48.6)	59 (54.1)	0.42
Serum amylase POD 1 [Umol/L] (IQR)	0.95 (0.23–2.42)	0.95 (0.24–2.5)	0.95 (0.23–2.34)	0.23
PPH [*n* (%)]	36 (16.4)	19 (17.3)	17 (15.5)	0.72
DGE [*n* (%)]	67 (30.5)	40 (36.4)	27 (24.5)	0.06
Rescue pancreatectomy [*n* (%)]	21 (9.5)	12 (10.9)	9 (8.2)	0.49
Surgical site infection [*n* (%)]	25 (11.4)	16 (14.5)	9 (8.2)	0.11
Readmission ICU [*n* (%)]	42 (19.2)	18 (16.5)	24 (21.8)	0.32
In-hospital mortality [*n* (%)]	12 (5.5)	10 (9.1)	2 (1.8)	**0.03**
Readmission rate [*n* (%)]	23 (16.2)	20 (18.2)	3 (9.4)	0.23
30-day readmission rate [*n* (%)]	19 (13.4)	16 (14.5)	3 (9.4)	0.45

*CR POPF* clinically relevant postoperative pancreatic fistula, *POH* postoperative hyperamylasemia, *PPAP* postpancreatectomy acute pancreatitis, *PPH* postpancreatectomy hemorrhage, *DGE* delayed gastric emptying, *POD* postoperative day, numbers in bold show significance if p<0.05

### Outcome analysis stratified by FRS

While stratifying the patient groups according to FRS in intermediate and high risk, results for postoperative morbidity still remained similar to the general outcome. The inhospital mortality was significantly higher in the non-HC group (9% vs. 1%, *p* = 0.03). The readmission rate (20% vs. 3%, *p* = 0.05) was also significantly higher in the non-HC group (20% vs. 3%, *p* = 0.05). Further, we observed more frequent events of PPH in the high-risk FRS receiving HC perioperatively, however, this just failed statistical significance (20% vs. 15%, *p* = 0.07) (Table 4).

**Table 4:** Sub-analysis in patients with intermediate or high risk of CR-POPF

	Intermediate -risk and high-risk		
	*Non-HC*	*HC*	*p *value
Patients *N* (%)	86	92	
Length of hospital stay (Days) (IQR)	27 (14–40)	26.5 (13–38)	0.53
Length of ICU stay (DAYS) (IQR)	3.8 (1.5–6.5)	8 (13–38)	0.54
Postoperative inhouse complication [*N* (%)]			
Complication [*N* (%)]	62 (72%)	69 (75%)	0.44
CDC ≥ 3 [*N* (%)]	37 (43%)	39 (42%)	0.28
CR-POPF [*N* (%)]	26 (30%)	28 (30%)	0.25
POH/PPAP [*N* (%)]	49 (57%)	58 (63%)	0.21
Serum amylase POD 1 [UMOL/L] (IQR)	1.49 (0.43–2.55)	1.37 (0.26–2.48)	0.07
PPH [*N* (%)]	17 (20%)	14 (15%)	0.07
DGE [*N* (%)]	33 (38%)	24 (26%)	0.10
Rescue pancreatectomy [*N* (%)]	11 (13%)	8 (9%)	0.20
Surgical site infection [*N* (%)]	9 (10%)	8 (9%)	0.26
Readmission ICU [*N* (%)]	15 (17%)	23 (25%)	0.30
In-hospital mortality [*N* (%)]	8 (9%)	1 (1%)	**0.03**
30-day readmission rate [*N* (%)]	15 (17%)	3 (3%)	**0.04**

## Discussion

Despite many years of research, morbidity after pancreatic surgery is still rather high and management may be challenging and prolonged. This can affect outcomes in patients with oncological findings, delaying adjuvant therapy due to a complicated postoperative course and reducing patients’ quality of life [11, 22]. In general, the treatment of complications in pancreatic surgery remains a problem, although the workup in high-volume centers has improved over the years [22]. An incomplete understanding of the pathophysiology of one of the major complications after PD - the POPF - may explain the lack of progress in this area. Recently, PPAP has gained attention and is increasingly considered to be a key player in triggering post-operative local inflammation and presumably POPF. It is known that major surgery can lead to both local and systemic inflammation, which may be the cause for both conditions [28].

**Fig. 2:**
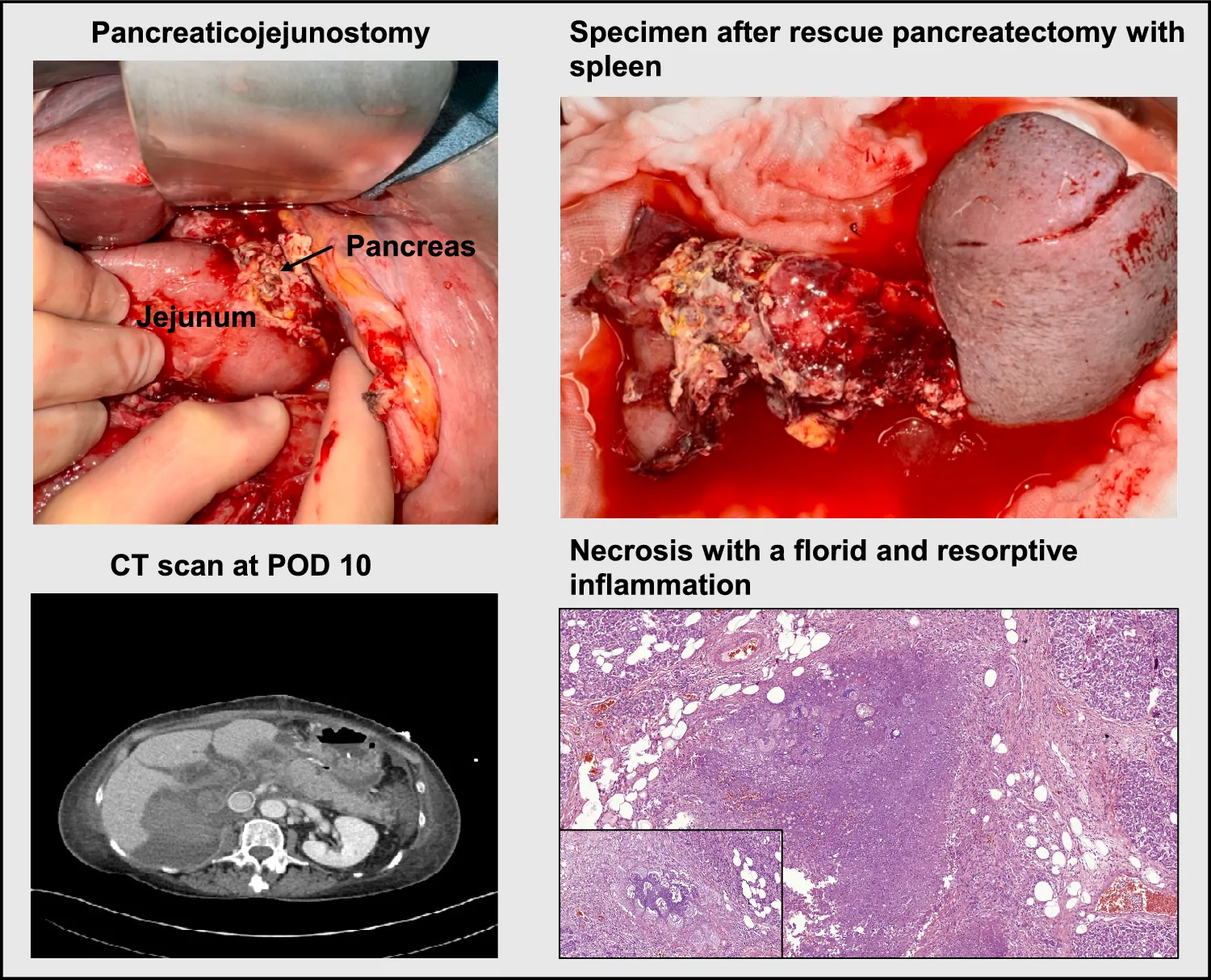
**a** an intraoperative image of a pancreaticojejunostomy after PD with pancreatitis. **b** specimen after rest (rescue) pancreatectomy with pancreatitis. **c** CT scan before rescue pancreatectomy with only limited changes for pancreatitis, differing from clinical presentation. **d** Pathological imaging of necrotizing pancreatitis with florid inflammation

A series of events related to surgical trauma, ranging from manipulation, mobilization, alteration of blood supply and/or stasis of pancreatic fluid, appears to be a central contributor in the development of PPAP (Fig. 2). Such pathophysiological conditions may trigger a cascade of acinar damage, cellular proteolytic enzyme activation, parenchymal oedema and peripancreatic inflammation, resulting in local and/or systemic effects. Thus, this inflammatory cascade in the early postoperative period may be a possible cause of further postoperative complications after PD, in particular POPF and PPAP [13].

The importance of postoperative hyperamylasemia on outcome after PD and distal pancreatic resection has been demonstrated in our previous studies. POH, as the early marker and part of the new ISGPS definition of PPAP, has a negative impact on postoperative morbidity [24, 25]. These results have since been analyzed by several authors and a negative impact of POH/PPAP on postoperative morbidity after pancreatic resection has been conformal reported by a majority of studies. In addition, POH/PPAP was also found to be an independent risk factor for POPF [1, 4, 16–18, 24, 25]. Alongside understanding the pathogenesis of postoperative inflammatory changes with the aim of both preventing their development and early recognition, prophylaxis seems to be attractive to enhance uneventfull postoperative recovery.

Steroids have a powerful inflammation-inhibiting ability that can reduce the inflammatory response after surgery. Some animal trials showed the importance of the acinar cells for the postoperative local inflammation, where prophylactic administration of corticoids perioperative was considered to reduce the severity of postoperative inflammation changes [7, 32]. However, a concern also remains that steroids may also lead to additional risk of anastomotic leakage and prolonged wound healing [23]. Nevertheless, the encouraging results from animal research and a meta-analysis have shown that steroids reduce the postoperative inflammatory response and postoperative complications [7, 20].

However, the limited data currently available from retrospective analyses, randomized controlled trials and meta-analyses is controversial. While some authors report on a significant effect of corticosteroids in reducing postoperative complications after pancreatic surgery, other fail to confirm their benefit perioperatively [2, 8, 12, 13, 27].

In the present study, we aimed to analyze the impact of HC on postoperative outcome while focusing on POPF and POH/PPAP. Our results showed no significant difference in postoperative complications associated with the administration of HC. To highlight these findings and allow comparability of patients in this retrospective study, we examined the results in a propensity score-matched cohort. In all procedure-specific complications, there was no significant difference after HC administration. These results are in line with currently published results of an RCT from China with 300 patients of which 134 received dexamethasone perioperatively [8]. The authors did not find a significant difference in postoperative complications and POPF in the dexamethasone group.

In our cohort, we observed a trend in the higher DGE rate, the prolonged hospital and ICU stay, as well as significantly increased mortality in the non- HC group. The more frequent occurrence of DGE might be a result of the significant higher rate of PPPD in this group. Among the ten patients who died in our study, eight in the non-HC group and two in the HC group developed septic complications of POPF and POH/PPAP causing in-hospital mortality. In the subanalysis of patients with intermediate and high FRS, mortality was even significantly higher in the non-HC group. Two reasons could explain this. The results may be subject to bias, as there were more patients with an ASA III score in the non-HC group. Furthermore, the majority of non-HC patients in this group were treated prior to 2019, before early completion of pancreatectomy became more frequent for septic complications postoperatively.

A Finish RCT analyzing patients after PD, who received HC perioperatively, shows significantly better results regarding postoperative complications (CDC ≥ 3), but not for POPF, PPH and DGE in the HC group. Patients were stratified according to the amount of acini on the resected pancreatic margin. Only the high-risk group received postoperative HC for 48 h. The authors conclude that HC may have an inhibitive effect on postoperative inflammation and impacts the outcome positively. However, the results for POPF did not show a significant difference in this trial, which is in line with our results [13]. Even after stratifying the patients according to FRS intermediate and high-risk groups, we could not find a significant difference in postoperative complications, rather a significantly high readmission rate and a significantly high rate of postoperative mortality in the non-HC group. The readmission rate was due to POPF Grade B, which was already analysed within the postoperative complications and didn`t show any significance between both groups even after stratifying to FRS. The possible reasons for the results of intraoperative mortality in the subanalysis were discussed above, interestingly the study by Chen et al. shows similar otcome [8].

The results of our study may add to the debate on the current limited data available concerning the benefits of corticosteroids in reducing postoperative complications. Nevertheless, it has several limitations. The first and most important one is the retrospective character of the study. Further, it is a single institution analysis and, therefore, prone to selection bias. Another important limitation, we consider, was the lack of information regarding the acines amount of the resection margin. For the analyzed cohort, this data was not available. We used FRS and the ISGPS ABCD risk classification for POPF to identify patients with high-risk anastomosis and stratified the results for these groups. However, certain biases may arise due to the similarity of the parameters, and we aim to address these within the limitations of the study.

However, this report gives an important insight into the real-world evidence on a propensity-score matched cohort. Our findings differ from these in the only two available HC RCTs, yet they appear to be similar to those of the dexamethasone RCT [8, 13]. Further prospective randomized trials are strongly required to evaluate the effect of corticoids on postoperative inflammation after PD and the potential benefit of their prophylactic administration perioperatively.

## Conclusion

The benefit of perioperative administration of HC for reducing postoperative complications following pancreatic resection cannot be demonstrated in this study. In particular, its anti-inflammatory effect did not impact the prevention of POH/PPAP. Further research is needed to understand if perioperative HC treatment is associated with potential benefits and especially its clinical effect on PPAP, based on the new classification of this complication.

## Data Availability

The original data in this study can be available from the corresponding author on reasonable request.
